# An Explainable Fusion of ECG and SpO_2_-Based Models for Real-Time Sleep Apnea Detection

**DOI:** 10.3390/bioengineering12040382

**Published:** 2025-04-03

**Authors:** Tanmoy Paul, Omiya Hassan, Christina S. McCrae, Syed Kamrul Islam, Abu Saleh Mohammad Mosa

**Affiliations:** 1 Department of Electrical Engineering and Computer Science, University of Missouri, Columbia, MO 65211, USA; tanmoy.paul@health.missouri.edu (T.P.); omiyahassan@boisestate.edu (O.H.); islams@missouri.edu (S.K.I.); 2 Department of Biomedical Informatics, Biostatistics, and Medical Epidemiology, School of Medicine, University of Missouri, Columbia, MO 65211, USA; 3 College of Nursing, University of South Florida, Tampa, FL 32603, USA; christinamccrae@usf.edu

**Keywords:** apnea, explainable AI, Grad-CAM, model fusion

## Abstract

Obstructive sleep apnea (OSA) is a common disorder characterized by disrupted breathing during sleep, leading to serious health consequences such as daytime fatigue, hypertension, metabolic issues, and cardiovascular disease. Polysomnography (PSG) is the standard diagnostic method but is costly and uncomfortable for patients, which has led to interest in artificial intelligence (AI) for automated OSA detection. To develop an explainable AI model that utilizes electrocardiogram (ECG) and blood oxygen saturation (SpO2) data for real-time apnea detection, providing visual explanations to enhance interpretability and support clinical decisions. It emphasizes giving visual explanations to show how specific segments of the signal contribute to the AI’s conclusions. Furthermore, it explores the combination of individual models to improve detection accuracy. The fusion of individual models demonstrates an enhanced performance in detection accuracy. Visual explanations for AI decisions highlight the importance of certain signal features, making the model’s operations transparent to healthcare providers. The proposed AI model addresses the crucial need for transparent and interpretable AI in healthcare. By providing real-time, explainable OSA detection, this approach represents a significant advancement in the field, potentially improving patient care and aiding in the early identification and management of OSA.

## 1. Introduction

Obstructive sleep apnea (OSA) is a commonly observed sleep-related breathing disorder resulting from the collapsing of the upper airway leading to disrupted airflow. This recurrent blockage causes breathing interruptions known as hypopnea and apnea, characterized by reduced airflow and a complete cessation of breathing for at least 10 s, respectively. Hypopnea is also marked by a decrease in blood oxygen levels by at least 4% [1,2,3]. Individuals experiencing moderate to severe apnea may encounter numerous such events during the night, leading to adverse health effects. The most prevalent consequence of OSA is daytime fatigue due to frequent awakenings [4]. Additionally, OSA is associated with elevated risks of high blood pressure, metabolic disorders, and cardiovascular diseases [5,6]. High-risk groups for OSA include patients with ischemic heart disease, heart failure, arrhythmias, cerebrovascular diseases, and type II diabetes [6,7,8]. Numerous studies have highlighted OSA as a risk factor for both pre- and post-surgery complications [9,10]. According to the American Academy of Sleep Medicine (AASM), approximately 5% of women and 14% of men in the United States are affected by sleep apnea, with a significant majority of cases going undiagnosed (around 80%) [11]. The estimated annual cost linked to undiagnosed sleep apnea is approximately USD 130 billion–USD 150 billion [11,12,13]. However, timely diagnosis of apnea has the potential to save up to USD 100.1 billion [11].

Polysomnography (PSG) conducted in a sleep laboratory stands as the predominant diagnostic approach for sleep apnea. This involves a patient spending a night or two in a sleep facility, where electrodes and wires are attached to record various physiological signals, including an electrocardiogram (ECG), electroencephalogram (EEG), electromyography (EMG), electrooculogram (EOG), blood oxygen saturation (SpO_2_), airflow, and respiratory effort [14,15]. PSG demands the presence of a sleep expert to monitor and analyze these signals, rendering it a time-consuming and costly procedure. The complex setup and discomfort caused by sensors may lead to the overestimation or underestimation of the severity of sleep apnea. Consequently, there exists a compelling need for an alternative to laboratory PSG that is more convenient and less intrusive. In the existing literature, numerous artificial intelligence (AI)-based detection techniques have been proposed as substitutes for polysomnography to facilitate the automated detection of obstructive sleep apnea.

Several studies have explored the use of individual biological markers, including SpO_2_, ECG, EOG, and EEG to identify apnea. The focus in many of these studies lies on SpO_2_ and ECG signals due to their correlation with apneic episodes. Apneic events typically result in blood oxygen desaturation, leading to an accelerated heart rate and increased systolic blood pressure [16]. To develop apnea detection models, various statistical, spectral, and nonlinear features are often extracted from the signals. Commonly utilized SpO_2_ features include the oxygen desaturation index, delta index, approximate entropy, Lempel–Ziv complexity, and central tendency measure, among others [17,18,19,20,21,22]. The prevalent features extracted from the ECG signal include instantaneous heart rate (IHR), ECG-derived respiration (ERD), and heart rate variation (HRV) [23,24]. However, manual feature engineering-based techniques demand for domain knowledge. Moreover, extracting meaningful features from noisy signals can be very difficult [25].

There are demonstrations of applying a wide array of classification models, such as logistic regression, AdaBoost (adaptive boosting), Naïve Bayes, k-nearest neighbor, multilayer perceptron, convolutional neural network (CNN), and long short-term memory (LSTM) network [26,27,28,29,30]. In general, the AI models work like black boxes where the reasoning behind the AI’s decision cannot be explained. Explainability in AI is crucial within the healthcare domain due to the critical nature of decision-making and the potential impact on patient outcomes. In healthcare, especially, there is a paramount need for transparency and interpretability in AI models to gain the trust of medical professionals, regulatory bodies, and patients. Clinicians and healthcare practitioners need to comprehend the rationale behind AI-driven recommendations or decisions to make informed choices in patient care. Explainability is essential for validating the reliability and safety of AI algorithms, ensuring that medical professionals can understand the underlying factors influencing the diagnostic predictions generated by the model. Additionally, in healthcare, where ethical considerations are paramount, explainability aids in identifying biases, errors, or potential pitfalls in AI algorithms, fostering accountability and facilitating continuous improvement in the deployment of these technologies for patient welfare. Ultimately, the integration of explainability in AI contributes to fostering a collaborative and ethical healthcare ecosystem. To the best of our knowledge, no existing studies provide explanations for CNN-based apnea detection using ECG and SpO_2_.

The objective of this study is to develop an explainable AI model for real-time apnea detection. The primary contributions of this study are as follows:

(1) The development of ECG and SpO_2_-based apnea detection models without manual feature extraction;
(2) A visual explanation of the models providing identification of the regions of the signal segments influencing the AI’s decision;
(3) Fusion of the individual models demonstrating the improvement of the overall performance of the individual models.

## 2. Materials and Methods

The ECG and SpO_2_ data utilized in this investigation were acquired from PhysioNet [31]. It is a repository of complex physiological signals, offering a comprehensive collection of data across various clinical domains, including sleep studies. Two distinct datasets were collected from PhysioNet, each detailed as follows:

Apnea-ECG Database [32]: This dataset encompasses a total of 70 ECG recordings and 8 SpO_2_ recordings. Collected from 32 subjects (25 males and 7 females, with an average age of 43 years), the recordings vary in duration from less than 7 h to nearly 10 h. The signals in this database are sampled at 100 Hz. The annotation scheme for the Apnea-ECG Database is minute-based, with each record segmented into non-overlapping one-minute intervals.

St. Vincent’s University Hospital Database [33]: The St. Vincent’s University Hospital Database consists of 25 complete overnight polysomnograms obtained from 21 male and 4 female subjects, with an average age of 50 ± 10 years (ranging from 28 to 68 years) and a mean body mass index (BMI) of 31.6 ± 40 kg/m^2^ (ranging from 25.1 to 42.5 kg/m^2^). The ECG signals in this dataset are sampled at 128 Hz, and the SpO_2_ signals are sampled at 8 Hz. The dataset follows a continuous annotation scheme, providing the onset time of sleep for each recording. Additionally, details regarding the onset time and duration of each apneic event are included.

### 2.1. Autocorrelation-Based Noisy ECG Segment Cancelation

The initial step involves segmenting each ECG and SpO_2_ recording into intervals of 11 s with overlap of 10 s. Subsequently, a bandpass Butterworth filter was applied to the ECG signal, with cutoff frequencies set at 1 Hz and 40 Hz. The choice of these frequency limits was guided by the low- and high-frequency parameters associated with a diagnostic ECG [34]. Additionally, the decision to exclude variations caused by baseline drifts also influenced the selection of the frequency bands. After filtering the signal segments, an autocorrelation-based noisy segment cancelation technique was applied, which was first proposed by Varon et al. [35]. In this technique, the autocorrelation function (ACF) of each segment was computed by taking the inverse of its power spectral density.

Following the computation of ACFs, the next step involved pinpointing the locations of contaminated segments. When an ECG is disrupted by artifacts, there is a discernible alteration in its autocorrelation. This signifies that the ACFs containing disturbances exhibit dissimilarity compared to the segments without noise. To identify these distinctions, the set of ACFs is graphically represented using a mathematical structure known as a graph. This graph comprises vertices, representing the ACFs, which are pairwise linked by edges corresponding to their similarity. The length of these edges was determined by the inverse of pairwise similarity between the ACFs, specifically employing cosine similarity as the quantifying metric.

(1) $$\cos\theta =\frac{A_{1}^{T}A_{2}}{\left\|A_{1}\right\|\left\|A_{2}\right\|},$$

In this context, $A_{1}$ and $A_{2}$ represent two vertices, with θ denoting the angle between them, $\left\|.\right\|$ defining the two-norm, and $A_{1}^{T}A_{2}$ indicating the dot product between the vectors. Consequently, segments exhibiting significant dissimilarities, such as artifacts, were depicted as isolated vertices in the graph. Each of these vertices was then characterized by a lower degree value, determined by aggregating the pairwise similarities between that specific vertex and the rest of the graph. When ECG segments were free from artifacts, their autocorrelation functions (ACFs) exhibit similarity, leading to high degree values for the corresponding vertices, indicating strong connectivity in the graph. These degrees were considered as “weights” to signify the cleanliness of a specific section in an ECG. Furthermore, the algorithm selectively preserves only the 95th percentile of the most analogous segments, aligning with the vertices possessing higher weights. In percentile analysis, the 95th percentile is a frequently employed threshold for identifying outliers.

### 2.2. Model Fusion Approach

**Figure 1:**
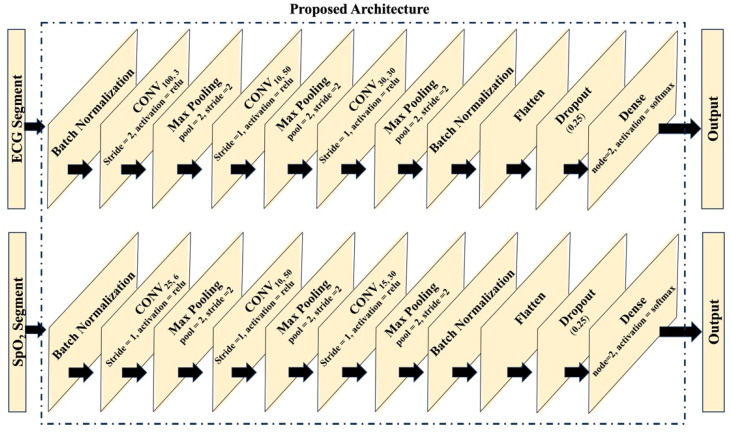
Architectures of the CNN models for individual signals.

Figure 1 shows the architecture of the individual signal-based models. In the ECG-based model, the input undergoes batch normalization, and there are three convolution layers (CONVs). These convolution layers have diverse configurations: the first convolution layer utilizes 3 kernels with a size of 100 and a stride of 2, the second convolution layer employs 50 kernels with a size of 10, and the third convolution layer integrates 30 kernels with a size of 30. Similarly, the SpO_2_ model follows a parallel architecture, utilizing three convolution layers. Specifically, the first convolution layer comprises 6 kernels with a size of 25, the second layer integrates 50 kernels with a size of 10, and the third layer encompasses 30 kernels with a size of 15. After each convolution layer, a maxpooling layer with a size and stride of 2 is implemented. Following the final maxpooling layers, flatten layers are used, accompanied by dropout layers with a ratio of 0.25. The output layers of both the ECG and SpO_2_ models adopt a dense configuration consisting of two neurons with softmax activation. It is noteworthy that all other layers within the architecture employ the rectified linear unit (ReLU) activation function.

**Figure 2:**
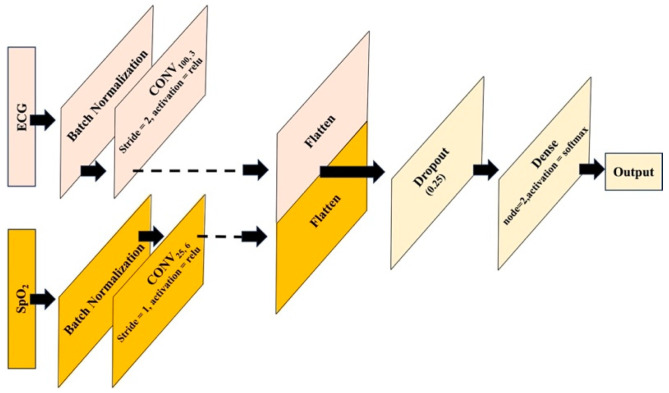
Architecture of the fusion-based model.

Once each independent model achieves satisfactory performance, the subsequent steps involve removing the output layers and concatenating the flatten layers. This combined layer is then connected to a fully connected dense network, followed by a final output layer to constitute the multi-sensor fusion model, as illustrated in Figure 2. The model operates on a feature-level fusion approach, utilizing a common dataset comprising diverse sensor signals for training. During training, all layers, excluding the final fully connected layer, remain frozen, akin to leveraging bottleneck features in transfer learning. The hyperparameters of the fully connected layers are fine-tuned for optimal performance on the fusion model using the validation set. Subsequently, the performance of the model is assessed on the test set.

The primary objective of the fusion algorithm is to enhance overall model performance. However, it is acknowledged that combining a well-performing model with a less effective one may occasionally result in lower performance for the fused model than for the individual models. Given that training is conducted solely on layers post the concatenated flatten stage, the network is expected to learn appropriate weights, discerning the reliability of each signal to enhance detection accuracies. This proves beneficial when individual models perform reasonably well, and amalgamating their learned features improves the overall model performance, constituting a data-driven fusion approach.

### 2.3. Selective Dropout

Consider a scenario where certain sensor sources exhibit higher sampling rates than others, which results in a greater number of data samples per second. In such situations, the decision-making of the fusion model becomes disproportionately influenced by the sensor source with a higher sampling frequency ($F_{s_{high}}$) compared to those with lower sampling frequencies ($F_{s_{low}}$). This situation can lead to the overfitting of the model to the data from the sensor source with the higher sampling frequency. The existing literature has discussed methods such as regularization and dropout to counter overfitting to the training data [36]. However, there is a gap in addressing overfitting to inputs from a single source in a multi-source input scenario.

In this context, a novel approach is proposed in this paper implementing selective dropout during the training phase at the flatten layer specifically for the features from the signal source with higher sampling rates. This ensures that the fusion model does not overfit to inputs with higher sampling rates. The advantage of this signal-based selective dropout lies in the fact that, during the inference stage, all features in the flatten stage can contribute to the fusion stage. This stands in contrast to undersampling the signal obtained at the higher sampling frequency, where dropped signals or features may not contribute to the model at all. Furthermore, the random nature of dropout during each training cycle guarantees that features at various positions contribute throughout the training process. This is distinct from undersampling, which often leads to the same samples or samples in fixed positions being dropped and not contributing to the fusion stage. Additionally, this proposed method represents the first endeavor to prevent overfitting in models with varying numbers of neurons in the flatten stage resulting from the optimization of individual model architectures.

For signals with different sampling frequencies but matching network structures, the dropout rate for the model associated with the signal having the higher sampling frequency should be set to $1-\frac{F_{s_{low}}}{F_{s_{high}}}$. Matched network structures imply that for two distinct signals with sampling rates $F_{s_{1}}$ and $F_{s_{2}}$ and a corresponding ratio of input window lengths $\frac{F_{s_{1}}}{F_{s_{2}}}$, the kernel sizes and number of neurons in each layer of the two networks should maintain the same $\frac{F_{s_{1}}}{F_{s_{2}}}$ ratio. In the event of a mismatch in network structure where the ratio of neurons in the output layer does not align with the ratio $\frac{F_{s_{1}}}{F_{s_{2}}}$, the dropout ratio ($D_{r}$) should be configured as follows:

(2) $$D_{r}=1-\frac{{neuron}_{s_{low}}}{{neuron}_{s_{high}}}$$

where ${neuron}_{s_{low}}$ denotes the number of neurons in the flatten layer of the model corresponding to the signal with the lower sampling frequency $F_{s_{low}}$ and ${neuron}_{s_{high}}$ represents the number of neurons in the flatten layer of the model associated with the signal having the higher sampling frequency $F_{s_{high}}$. In situations where the fusion involves k different sensors operating at k different sampling frequencies, the dropout ratio $D_{r}$ for each $j^{th}$ signal model can be computed as follows:

(3) $$D_{r,j}=1-\frac{\min\left(F_{s_{1}},F_{s_{2}},\ldots ,F_{s_{k}}\right)}{F_{s_{j}}}\forall j\in [1,\ldots ,k]$$

### 2.4. Evaluation with Noisy Data

To assess the efficacy of fusion models in the presence of noise, simulations involving scenarios with noisy signal windows were conducted, where all samples within a noisy signal window were corrupted by noise. This was achieved by introducing −20 dB white Gaussian noise to the signal segments. The use of white Gaussian noise was chosen to encompass all noise frequencies, despite the prevalence of low-frequency noises during sleep. According to the central limit theorem, the sum of multiple independent distributions tends to form a Gaussian distribution. Therefore, white Gaussian noise was introduced to the signals to investigate model performance in noisy scenarios.

For both the training and the validation sets, 20% of signal samples within a window in the ECG window set were made noisy, while the corresponding SpO_2_ window set remained clean. Similarly, 20% of signal samples within a window in the SpO_2_ window set were rendered noisy, while the corresponding ECG window set remained clean. Additionally, 20% of signal samples within a window in both the SpO_2_ and ECG window sets were made noisy for both apnea and non-apnea events. In the test set, 11.42% of signal samples within a window in the ECG window set were subjected to noise, while the corresponding SpO_2_ window set remained noise-free. Likewise, 11.42% of signal samples within a window in the SpO_2_ window set were exposed to noise, while the corresponding ECG window set remained clean. Furthermore, 11.42% of signal samples within a window in both the SpO_2_ and ECG window sets were subjected to noise for both apnea and non-apnea events.

### 2.5. Visual Explanation of the Model

Class activation mapping (CAM) is a gradient-based technique developed to improve the comprehension of convolutional neural network (CNN) model predictions, especially in image analysis tasks. CAM utilizes global average pooling within the layers of a convolutional network to compute class activation maps. This pooling operation is pivotal in identifying specific regions within an image that significantly contribute to explaining the predictions generated by the model. Through CAM, one can effectively identify crucial image regions by projecting the weights of the models onto the convolutional feature maps. It is noteworthy that CAM requires a specific CNN architecture, one that excludes fully connected layers. Gradient-weighted class activation mapping (Grad-CAM) represents a notable advancement beyond CAM, addressing its limitation of relying on a particular CNN architecture. What sets Grad-CAM apart is its remarkable ability to generate explanations for any CNN-based network without requiring modifications to the architecture of the network. This makes Grad-CAM highly versatile and adaptable which is applicable to a diverse range of CNN models. Its distinctiveness lies in its discriminative power, leveraging gradients associated with any class concept to yield insightful explanations.

Grad-CAM begins by computing the gradient of the class score, denoted as y^c^, with respect to a specific class c. This gradient calculation is performed in relation to the feature map A^k^, which resides in a spatial dimension of H (height) by W (width) and belongs to channel k. Following the gradient calculation, a global average pooling operation is applied to this gradient. This pooling operation summarizes the gradient information across the spatial dimensions, effectively aggregating the gradient values as shown in Equation (4).

(4) $$a_{c}^{k}=\frac{1}{Z}\sum_{i}\sum_{j}\frac{\partial y^{c}}{\partial A_{ij}^{k}}$$

In the context of this equation, where Z represents the product of the height (H) and the width (W) of the feature map, and *i* and *j* denote individual pixels within the feature map, the calculated weight $a_{c}^{k}$ Is determined by Equation (4), which quantifies the significance of a feature contained within the feature map A_k_ in relation to the prediction y^c^. Subsequently, in the Grad-CAM method, a weighted summation is performed, combining α_c_^k^ and the corresponding feature map A^k^. This summation is followed by the application of the rectified linear unit (ReLU) function, which effectively eliminates negative values. The resulting output, denoted as L_c_, takes the form of a heatmap. This process results in a heatmap that highlights the significant pixels or regions within the image that contribute most to the classification of the relevant concept or class *c*. These highlighted regions serve as valuable visual explanations, aiding in the interpretation of the decision-making process of the CNN. Grad-CAM is a powerful and versatile technique for generating explanations in CNN-based models, allowing one to gain insights into which image regions are influential in making specific predictions. Unlike its predecessor CAM, Grad-CAM can be applied to a wide array of CNN architectures without requiring any architectural changes, making it a valuable tool for interpretability and visualization in deep learning applications, particularly in image analysis tasks.

(5) $$L^{C}=RELU(\sum_{k}a_{c}^{k}A^{k})$$

## 3. Results

Table 1 presents a breakdown of the distribution of 11 s signal segments obtained by aggregating two datasets. A class imbalance emerged after segmenting the signals, with nearly 80% of ECG signal segments and around 91% of SpO_2_ signal segments falling into the normal class. The signal segments were split into train, test, and validation sets with a ratio of 8:1:1. To address this significant imbalance, random oversampling was employed on the training set, followed by an augmentation technique that involves flipping the segments effectively doubling the overall number of segments. The table provides insights into the count of signal segments within the training, validation, and test sets. Upon closer inspection, it becomes apparent that the number of segments derived from the SpO_2_ signal was lower than that from the ECG signal. This disparity is attributed to the Apnea-ECG dataset containing only 8 SpO_2_ recordings compared to the 70 ECG recordings, resulting in a smaller number of SpO_2_ segments.

**Figure 3:**
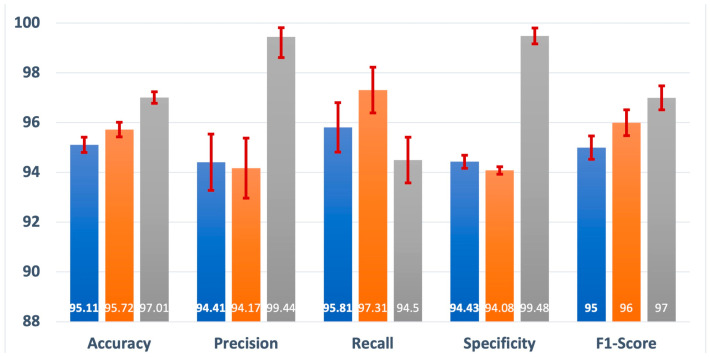
Performance of the individual signal-based models and fusion-based model and associated confidence interval.

A comparison of the performance of the individual signal-based models against the fusion-based model is presented in Figure 3. The effectiveness of these models in detecting apnea was evaluated using multiple performance metrics, including accuracy, precision, recall, specificity, and F1-score. Interestingly, both the ECG and the SpO_2_-based models exhibited similar performance across all the assessed metrics, indicating a comparable ability to identify instances of apnea. The ECG signal-based model demonstrated an accuracy of 95.11%, precision of 94.41%, recall of 95.81%, specificity of 94.43%, and an F1-score of 95. On the other hand, the SpO_2_ signal-based model achieved an accuracy of 95.72%, precision of 94.17%, recall of 97.31%, specificity of 94.08%, and an F1-score of 96. However, the standout observation arises when comparing the performance of the individual models with that of the fused model. The fusion-based model exhibited superior performance, particularly evident in its precision of 99.44% and specificity of 99.48%. These findings suggest that the combination of ECG and SpO_2_ signals in the fused model enhances the overall accuracy and precision in detecting apnea, surpassing the capabilities of the individual signal-based models.

**Figure 4:**
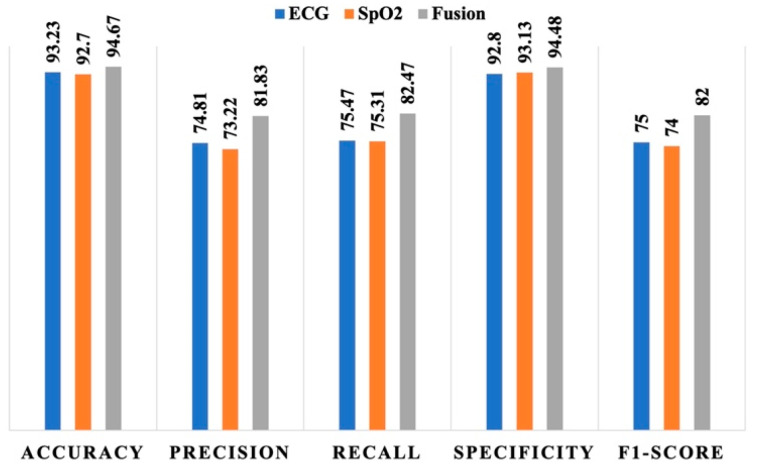
Performance of the individual signal-based models and fusion-based model with added noise.

The analysis of how the models perform in predicting apnea from noisy segments is depicted in Figure 4. The influence of noisy segments is evident as it impacts the performance of all models. While the accuracy and specificity of the models show no significant decrease, the precision and recall values are notably lower compared to those illustrated in Figure 3. For the noisy segments, the ECG-based model demonstrated a precision of 74.81%, a recall of 75.47%, and an F1-score of 75. Conversely, the SpO_2_-based model exhibited precision, recall, and F1-score of 73.32%, 75.31%, and 74. A crucial observation from this analysis is that the fusion-based model outperformed the individual signal-based models significantly when additional noise was introduced in the signal segments. The fusion-based model yielded a precision, recall, and F1-score of 81.83%, 82.47%, and 82, respectively, highlighting its enhanced performance under conditions of increased noise.

**Figure 5:**
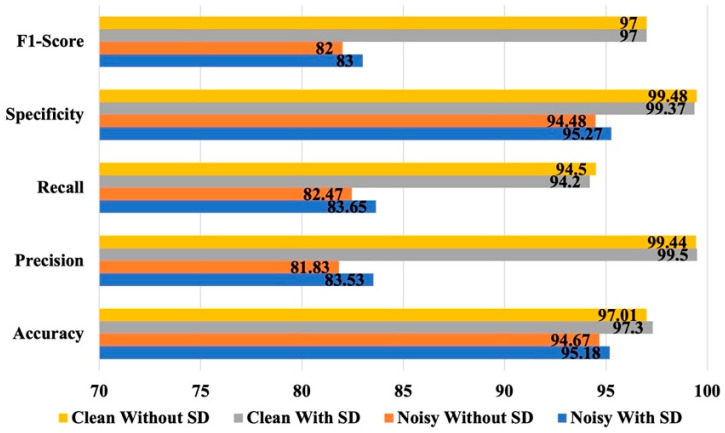
Effect of selective dropout (SD) on the fusion-based model.

The efficacy of the selective dropout technique is presented in Figure 5. It is evident that the application of selective dropout did not have any discernible effect on the performance of the models for the clean signal segments. The values of the performance metrics were almost same for the clean segments. Interestingly, for the noisy segments, the application of selective dropout had a small effect on the performance of the model. The model with selective dropout performed slightly better than the other model. For the noisy segments, the model without selective dropout yielded an accuracy of 94.67%, a precision of 81.83%, a recall of 82.47%, a specificity of 94.48%, and an F_1_-score of 82. On the other hand, application of selective dropout resulted in improved apnea detection with 95.18% accuracy, 83.53% precision, 83.65% recall, 95.27% specificity, and 83 F_1_-score.

**Figure 6:**
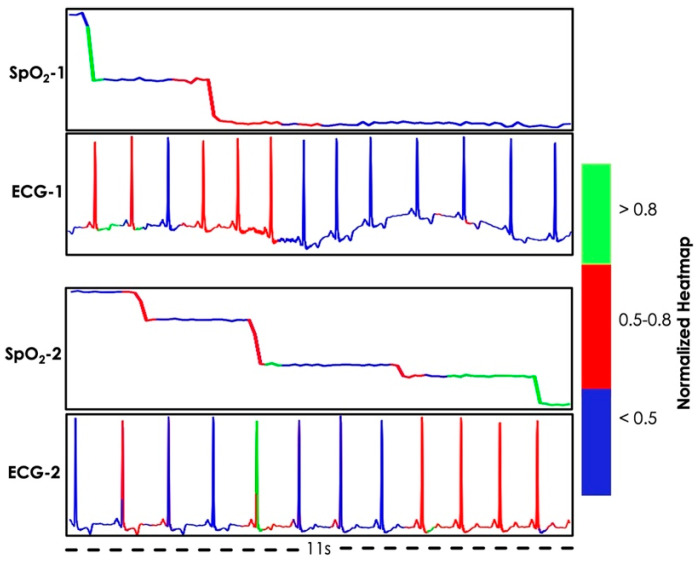
Grad–CAM generated heatmap.

Figure 6 demonstrates the explainability of the proposed individual signal-based model by illustrating the heatmap generated by Grad-CAM. The objective of using Grad-CAM was to inspect which region of the input the model gives more importance in its decision-making. Furthermore, the goal was to examine whether that reasoning is consistent with human clinical understanding. Here two random pairs of apneic segments of SpO_2_ and corresponding ECG signals are shown. According to visual inspection, the apneic activity should be determined based on the oxygen desaturation region and corresponding ECG region. An explainable model should focus more on this region while making its decision. The generated heatmap was normalized and quantized with specific thresholds as shown in the color bar in Figure 6. The green and blue color signifies the highest and the lowest importance, respectively and the red color denotes moderate importance. In addition, it can be seen that the activation heatmap of the model is consistent with our visual inspection. For both pairs, the models put moderate to high importance on the desaturation region of the SpO_2_ segment and the corresponding ECG segment.

## 4. Discussion

Table 2 presents a comparative analysis between the proposed CNN architectures for ECG and SpO_2_ signals and several existing studies. The comparison includes various aspects such as processing window duration, data preprocessing methods, input format, classifier type, and key performance metrics (accuracy, precision, recall, specificity, and F1-score). A detailed examination of the results shows that the proposed models generally outperform prior approaches across most evaluation metrics [26,27,37,38]. In cases where other models achieved slightly better results, they often relied on computationally intensive preprocessing steps or used longer processing windows, making them less suitable for real-time applications due to increased inference time and resource demands.

The identification of apneic activities during sleep is a complex task that cannot be solely reliant on a single physiological marker. While it is true that the heart rate tends to increase during apneic events, using heart rate alone as the sole biological marker for apnea detection is not sufficient. This limitation arises because several other physiological factors can influence heart rate fluctuations, and not all variations in heart rate are indicative of apnea. Therefore, it is essential to consider multiple physiological signals to enhance the accuracy of apnea detection. Similarly, relying solely on oxygen desaturation events as a single marker for apnea detection is also inadequate. Oxygen desaturation can occur for various reasons other than apnea, and it may not always coincide with apneic events. Recognizing the complexity of sleep-related disorders, the American Academy of Sleep Medicine (AASM) recommends the use of apnea monitoring devices that incorporate multiple physiological channels for a more comprehensive assessment. This study proposes a fusion-based model where the decision is driven by both ECG and SpO_2_ segments.

AI-driven apnea detection systems offer a multitude of benefits across different healthcare environments. In sleep clinics, AI-powered apnea detection systems automate the labor-intensive process of sleep study analysis. This automation not only saves valuable time but also significantly improves the accuracy of the identification of apnea events. Instead of healthcare professionals manually reviewing hours of recorded sleep data, AI algorithms can swiftly and precisely identify apnea events. This, in turn allows clinicians to redirect their focus towards interpreting the results and tailoring appropriate treatment plans for patients. In hospital settings, AI algorithms play a vital role in continuously monitoring patients who are at risk of apnea. These algorithms can promptly detect apnea episodes enabling timely medical intervention. Real-time monitoring enhances patient safety, particularly among individuals recovering from surgery or residing in critical care units. The early detection and intervention capabilities of AI-driven systems help reduce the risk of complications associated with apnea ensuring better patient outcomes. Additionally, wearable devices equipped with AI technology extend the reach of apnea detection into home-based care. These devices can track sleep patterns and detect apnea events in the comfort of the patient’s home. This not only facilitates remote monitoring but also allows for personalized interventions based on the collected data. Overall, AI-driven apnea detection systems offer a multifaceted enhancement to healthcare by improving diagnostic efficiency, enhancing patient safety, and increasing accessibility to apnea management. This transformative technology is revolutionizing how healthcare providers diagnose and manage apnea, ultimately leading to better patient care.

The fusion algorithm aims to enhance overall model performance, but in some cases, combining a well-performing model with a less effective one might result in lower performance for the fused model compared to individual models. By freezing initial layers and training only on layers post the concatenated flatten stage, the network is designed to learn appropriate weights, prioritizing signals for improved detection accuracy. This proves beneficial when individual models already perform reasonably well, allowing the fusion of information from features learned by each model to follow a data-driven approach, enhancing the performance of the fused model. This proves advantageous in wearable devices, where individual sensor features in the flatten stage can be employed for single-sensor source inference, either through fusion or utilizing the best-performing sensor based on battery levels. This flexible approach requires minimal additional memory or area while the training time of the fused model is reduced ensuring confidence in its performance matching or surpassing individual models.

## 5. Conclusions

In conclusion, this research underscores the critical need for efficient and accessible methods of obstructive sleep apnea detection, given its widespread prevalence and associated health risks. Polysomnography, the current diagnostic standard, presents challenges in terms of resource intensity and patient discomfort. The exploration of AI as an alternative for automated OSA detection aligns with the demand for more convenient and less intrusive diagnostic approaches. Our study focuses on developing an explainable AI model for real-time apnea detection, leveraging ECG and SpO_2_ signals. By proposing apnea detection models without manual feature extraction and offering visual explanations of AI decisions, this research contributes to the interpretability and transparency of healthcare AI models which is a crucial factor in gaining trust from medical professionals, regulatory bodies, and patients. The demonstrated fusion of individual models further enhances overall performance, emphasizing the potential of AI-driven solutions in improving patient outcomes. This study represents a significant step forward in the pursuit of efficient and transparent AI applications in healthcare, particularly in the realm of sleep apnea detection.

## Figures and Tables

**Table 1:** Distribution of signal segments with the processing window of 11 s.

	ECG			SpO_2_		
	Train	Validation	Test	Train	Validation	Test
Total	214,264	8267	8264	152,364	5216	5222
Apnea	107,132	1572	1570	76,182	456	460
Normal	107,132	6695	6694	76,182	4760	4762

**Table 2:** Performance comparison with state-of-the-art studies.

Signal	Study	Processing Window (s)	Data Cleaning	Model Input	Classifier	Accuracy (%)	Precision (%)	Recall (%)	Specificity (%)	F_1_-Score
ECG	Wang et al. [26]	300	Band-pass Filter	R-R Interval	CNN	87.6	N/A	83.1	90.3	N/A
	Bai et al. [27]	60	Encoder-Decoder	R-R Interval	CNN	92.0	N/A	90.0	N/A	N/A
	Zarei et al. [28]	60	Band-pass Filter	Filtered Signal	CNN + LSTM	97.21	N/A	94.41	98.94	N/A
	Urtnassan et al. [37]	10	Band-pass Filter	Filtered Signal	CNN	N/A	96	96	N/A	0.96
	***Proposed Model***	***11***	***N/A***	***Raw Signal***	***CNN***	***95***	***94***	***95***	***94***	***0.95***
SpO_2_	Mostofa et al. [38]	60–300	Resampling	Resampled Signal	CNN	94	N/A	92	96	N/A
	Ma et al. [39]	60	Artifact Rejection	Extracted Feature	SVM	90.2	N/A	87.6	94.1	N/A
	Lyden et al. [40]	60	N/A	LSTM extracted features	Naïve Bayes	97.04	97.19	96.94	N/A	N/A
	***Proposed Model***	***11***	***N/A***	***Raw Signal***	***CNN***	***95***	***94***	***97***	***94***	***0.96***

## Data Availability

The dataset used in this article is available in the Physionet Repository (https://physionet.org/), which is a public resource for research data (accessed on 10 January 2023).
